# Anticancer effects of metformin in experimental animal models of different types of cancer: a systematic review and meta-analysis

**DOI:** 10.1186/s42826-022-00131-6

**Published:** 2022-07-19

**Authors:** Fan Zhang, Shuai Han, Weijie Song

**Affiliations:** 1 Tianjin Medical University Cancer Institute and Hospital, National Clinical Research Center for Cancer, Key Laboratory of Cancer Prevention and Therapy, Tianjin, 300060 People’s Republic of China; 2 Experimental Animal Center of Inner Mongolia Medical University, Inner Mongolia Medical University, Hohhot, 010050 Inner Mongolia People’s Republic of China

**Keywords:** Metformin, Animal models, Cancer, Meta-analysis

## Abstract

To systematically evaluate the effects of metformin on tumors in experimental animal models of different types of cancer. Pubmed, Embase, Cochrane, and Web of Science databases were searched for studies on metformin used in various experimental animal tumor models from 2008 to 2022. Meta-analysis was performed using STATA 16.0 software after screening literature extraction data and methodological quality evaluation by inclusion and exclusion criteria. A total of 24 studies with 1108 model animals were included. Meta-analysis results showed that this study used meta-analysis for quantitative synthesis of study results and found that tumor model animals of different species showed different degrees of reduction in tumor volume, weight, and number after metformin intervention.

## Background

Metformin can reduce fasting and postprandial glucose in diabetic patients by reducing hepatic glucose synthesis and intestinal glucose absorption and is mainly used in obese type 2 diabetes [1, 2]. Recent studies have found that metformin can also inhibit the proliferation, migration, and invasion of tumor cells, inhibit tumor angiogenesis, interfere with the tumor cell cycle, and induce apoptosis, cell differentiation, and other antitumor effects [3].

However, there is little clinical evidence to guide the use of metformin as an antitumor agent in patients with cancer. And are there differences in the tumor-suppressive effects of metformin in patients with different tumor types? In order to study the tumor suppressive effect of metformin, many researchers have established animal models of different tumors to verify the mechanism of metformin cancer inhibition. However, due to various conditions, many independent studies have imperfections (e.g., too small sample size, narrow age range coverage, gender imbalance, or inability to obtain richer and more accurate results due to the limitations of experimental techniques at that time). Therefore, the use of systematic reviews and meta-analyses to integrate the results of multiple studies allowed for larger data samples and improved the precision and accuracy of the results. The results of the analysis will help elucidate whether animal models of different types of cancer can benefit from metformin treatment and help translate animal studies into clinical research.

## Materials and methods

### Literature search and inclusion criteria

Keywords published in Pubmed, Embase, Cochrane, Web of science from 2008 to 2022 were searched, including "cancer," "oncology," "metformin," "experimental animals." "metformin," "experimental animals," and we did not set language restrictions. Inclusion criteria were: 1. studies of the anticancer effects of metformin; 2. studies using animal models; 3. studies reporting at least one outcome indicator related to antitumor effects. In vitro studies and studies on human participants were excluded. Duplicate studies conducted by the same authors will not be included. Two authors independently reviewed the titles and abstracts identified in the search. During this process, articles were discussed, and after excluding those that did not meet the inclusion criteria, the two reviewers read the remaining articles in their entirety to ensure that they truly met the inclusion criteria. If the issue remained unresolved, a third assessor was asked to make a decision. Any disputes were resolved by discussion with the third reviewer to reach a consensus among all reviewers.

Retrieve expression (Pubmed): (((("Neoplasms"[Mesh]) OR (((((((((((((((((Tumor[Title/Abstract]) OR (Neoplasm[Title/Abstract])) OR (Tumors[Title/Abstract])) OR (Neoplasia[Title/Abstract])) OR (Neoplasias[Title/Abstract])) OR (Cancer[Title/Abstract])) OR (Cancers[Title/Abstract])) OR (Malignant Neoplasm[Title/Abstract])) OR (Malignancy[Title/Abstract])) OR (Malignancies[Title/Abstract])) OR (Malignant Neoplasms[Title/Abstract])) OR (Neoplasm, Malignant[Title/Abstract])) OR (Neoplasms, Malignant[Title/Abstract])) OR (Benign Neoplasms[Title/Abstract])) OR (Benign Neoplasm[Title/Abstract])) OR (Neoplasms, Benign[Title/Abstract])) OR (Neoplasm, Benign[Title/Abstract]))) AND (("Metformin"[Mesh]) OR (((((((Dimethylbiguanidine[Title/Abstract]) OR (Dimethylguanylguanidine[Title/Abstract])) OR (Glucophage[Title/Abstract])) OR (Metformin Hydrochloride[Title/Abstract])) OR (Hydrochloride, Metformin[Title/Abstract])) OR (Metformin HCl[Title/Abstract])) OR (HCl, Metformin[Title/Abstract])))) AND (("Mice"[Mesh]) OR ((((((((((((((((((Mus[Title/Abstract]) OR (Mouse[Title/Abstract])) OR (Mus domesticus[Title/Abstract])) OR (Mus musculus domesticus[Title/Abstract])) OR (domesticus, Mus musculus[Title/Abstract])) OR (Mus musculus[Title/Abstract])) OR (Mice, House[Title/Abstract])) OR (House Mice[Title/Abstract])) OR (Mouse, House[Title/Abstract])) OR (House Mouse[Title/Abstract])) OR (Mouse, Swiss[Title/Abstract])) OR (Swiss Mouse[Title/Abstract])) OR (Swiss Mice[Title/Abstract])) OR (Mice, Swiss[Title/Abstract])) OR (Mice, Laboratory[Title/Abstract])) OR (Laboratory Mice[Title/Abstract])) OR (Mouse, Laboratory[Title/Abstract])) OR (Laboratory Mouse[Title/Abstract])))) AND (randomized controlled trial OR randomized OR placebo).

### Quality assessment and data extraction

The methodological quality of the included studies was evaluated according to Camarades' criteria, which consisted of 10 items: publication after peer review, temperature control of experimental animals, random assignment, blinding to animal model induction, blinding to outcome assessment, reasonable application of animal anesthetic drugs, appropriate animal models, sample size calculation, compliance with animal protection laws, and declaration of potential conflicts of interest. The total score for the evaluation criteria was 10 [4].

### Data analysis

Meta-analysis was performed using the RevMan 4.2.2 software provided by the Cochrane Collaboration Network. Statistical heterogeneity was first tested by q test with the test level set at α = 0.05, and then studies with no heterogeneity (P > 0.05) were analyzed using a fixed-effects model, and studies with heterogeneity (P < 0.05) were analyzed using a random-effects model. Effect sizes were combined using the relative ratio (OR) and its 95% confidence interval (95% CI) for categorical variables and mean difference (MD) and its 95% CI for continuous variables, and sensitivity analysis was performed on the relevant results when necessary.

Chi-square test and I^2^ analysis were applied to test for heterogeneity among the included studies. The I^2^ value describes the percentage of inter-trial variation due to heterogeneity (rather than randomness) among the pilot studies included in the meta-analysis as a percentage of the total variation. When the final analysis resulted in P < 0.05 (I^2^ > 50%), significant heterogeneity between groups was indicated; if the analysis resulted in P ≥ 0.05 (I^2^ ≤ 50%), no heterogeneity between studies was indicated.

Funnel plots are mainly used to observe whether there is bias, such as publication bias or other bias, in the results of a particular systematic evaluation or Meta-analysis. If there is bias in the information, an asymmetric funnel plot will appear, and the more pronounced the asymmetry, the greater the degree of bias. The asymmetry of the funnel plot is mainly related to publication bias, but other reasons may also exist. In this study, the funnel plot drawn by RevMan 4.2.2 was used to analyze publication bias.

## Results

### Search results

**Fig. 1:**
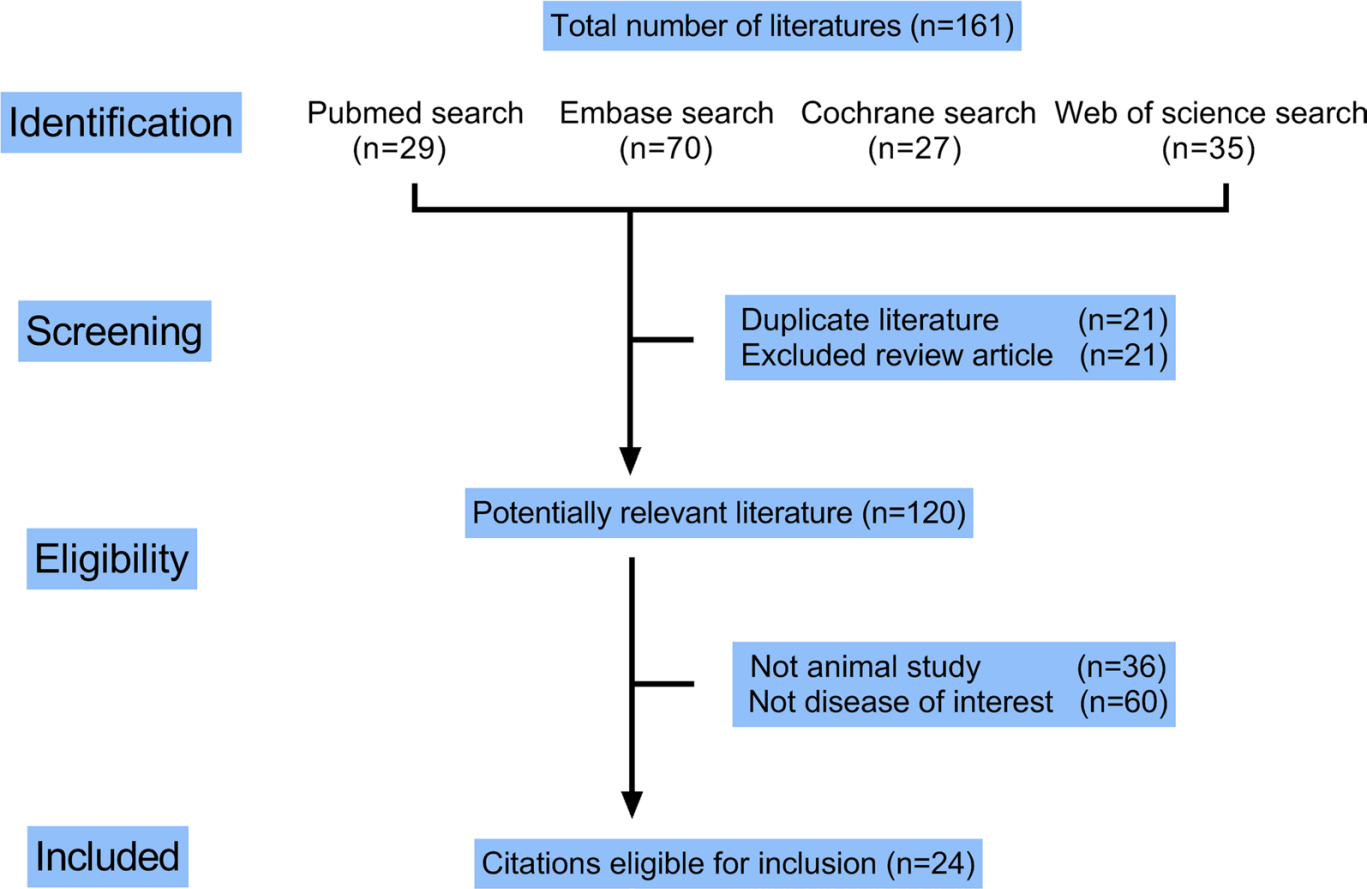
Flow chart for the selection of records to include

A total of 969 potential articles were identified through the literature search. After screening, 161 articles were included in the screening, and the articles were from Pubmed (n = 29), Embase (n = 70), Cochrane (n = 27), and Web of science (n = 35). Among them, 21 duplicate articles, 21 review articles, 13 non-animal experiment articles, and 60 non-relevant articles of this study were excluded, after which 120 potentially relevant articles remained, and 24 articles were screened for our meta-analysis. The flowchart in Fig. 1 shows the selection process. A review of study selection and data extraction showed excellent inter-rater agreement. Study characteristics are summarized in Table 1.

**Table 1:** Characteristics of references enrolled in this Meta-analysis

Author	Year	Cancer types	Species, strain	Moulding method	Dosage	Adnimistration	Outcome	Score
Wu.S	2014	Lung Carcinoma	C57BL/6 J,M,5-6w	LLC cells	–	–	TV	7
			Wistar rats, F, 5w	MNU induces	–	–	TV	8
Algire.C	2008		SCID mice, F, 4-5w	MCF10AT cells	50 mg/kg	p.o	TV	7
Seabloom.D	2015		Wistar rats	DEN induces	12 mg/g	p.o	TN	7
Zhou.X	2017	Hepatocellular Carcinoma	SHR mice, F,6-8w	Fluka induces	50 mg/kg	p.o	TV	5
Tijeras-Raballand.A	2020		SHR mice, F,6-8w	Fluka induces	150 mg/kg	p.o	TN	6
D.K. DePeralt	2014		C57BL/6 mice	melanoma cells SK-23	50 mg/kg	i.p	TW	6
Hsueh.E. C	2013	Melanoma	BALB/c nude	SW480 cells	250 mg/kg	p.o	TV	9
Takahashi.H	2013	Colon Cancer	athymic mice	LNCaP cells	250 mg/kg	p.o	TV	6
Madka.V	2019		C57BL/6 J mice, F, 6-8 W	OvCa cells	70 mg/kg	p.o	TW	6
Litchfield.L. M	2014	Ovarian cancer	nude mice	human pancreatic cancer cells	250 mg/kg	p.o	TV	7
Mills.K. A	2015		athymic nude mice	SKOV3ip1 cells	200 mg/kg	p.o	TN	8
Lengyel.E	2015		F344 rat, M,4-6w	azoxymethane-induced	250 mg/kg	i.p	TN	6
Deriabina.O.N	2010	Skin Carcinoma	nude mice	PDX	200 mg/kg	p.o	TV	6
			nu/nu athymic mice, F, 6w	PDX	-	-	TV	7
Wei.L	2019	Prostate cancer	A/J mice, F, 7w	transgenic animal	100 mg/kg	i.p	TN	5
Hou.M	2010		nude mice	CFPAC-1 cell line	50 mg/kg	p.o	TV	6
Cufí.S	2013	Mammary tumor	ApcMin/ + mice	transgenic animal	250 mg/kg	p.o	TN	8
Checkley.L.A	2017		C57BL/6, F, 5-6w	RENCA cells	70 mg/kg	p.o	TV	7
Takahashi.M	2015	Renal carcinoma	LSL-KrasG12D/ + ;Trp53F2-10mice	transgenic animal	50 mg/kg	p.o	TV	6
Rajeshkumar.N.V	2017	Pancreatic Cancer	nude mice, M, 4-6w	HepG 2 cells line	250 mg/kg	i.p	TV	8
Kisfalvi.K	2009		C57BL/6 mice, M, 6w	pancreatic cancer cells	250 mg/kg	p.o	TW	7
Cifarelli.V	2015		C57BL/6, M, 4-8w	RM-1 cells	250 mg/kg	p.o	TV	6
Tan.X. L	2015		C57BL/6	LLC cells	250 mg/kg	p.o	TN	7
Shi.Y. Q	2016		nude mice, M, 4-6w	HepG 2 cells line	200 mg/kg	p.o	TV	8
Fernandes.J.M	2019	Rectal Cancer	LKB1fl/flp53fl/fl mouse	ECC-1 and Ishikawa cells line	250 mg/kg	i.p	TW	6

### Risk of bias and quality of included studies

**Fig. 2:**
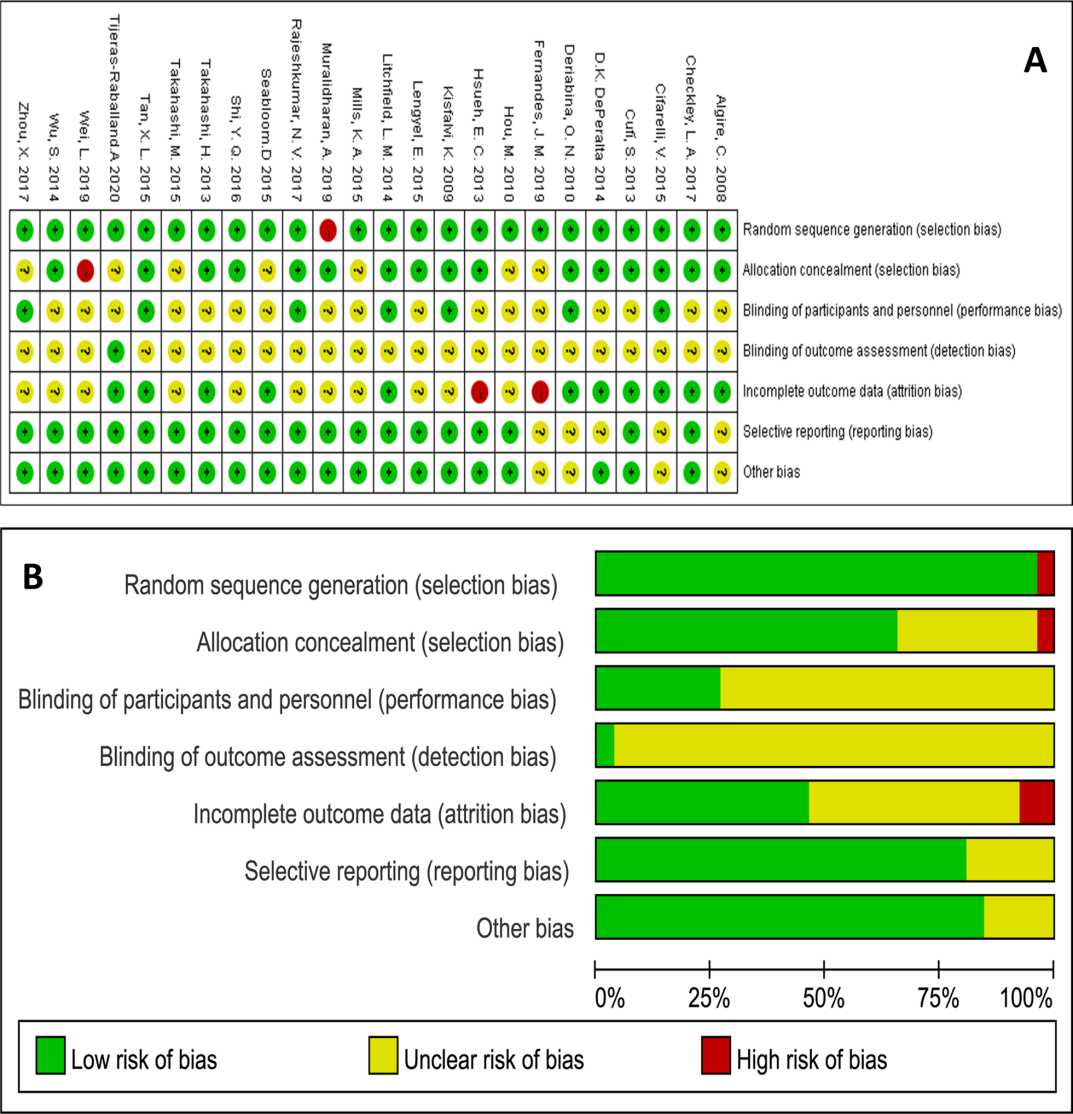
Risk of bias graph. **a** Review authors’ judgements about each risk of bias item for each included study. **b** Review authors’ judgements about each risk of bias item presented as percentages across all included studies

The final 24 included papers were all randomized controlled trials, and analysis for the 7 risk of bias assessment criteria in Revman showed that 18 (75%) papers had 4 or more low-risk items. And most studies did not describe the method of randomization because the background of the experimental animals included in each study was essentially homogeneous. The risk of bias summary and risk of bias graph is shown in Fig. 2a, b.

### Overall analysis of the effect of metformin on the growth of various types of tumors

**Fig. 3:**
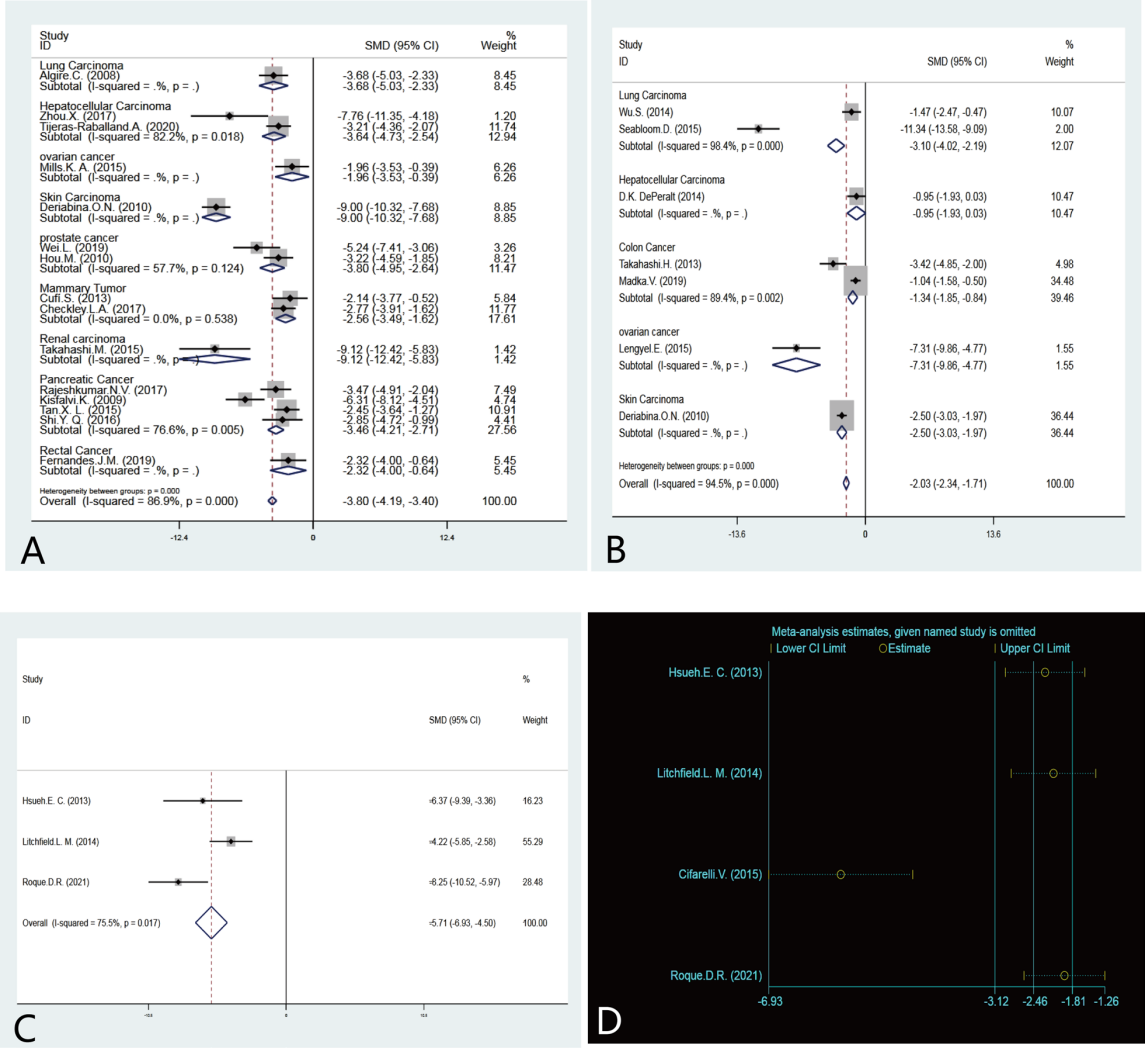
Forest plot of the effect of metformin on tumor growth in different types of animal models. **a** Forest plot of the tumor volume. **b** Forest plot of the tumor number. **c** Forest plot of the tumor weight. **d** Metaninf plot of the tumor weigh

The 15 papers included in the current study reported 724 experimental animals, which involved 9 different cancer studies, namely Lung Carcinoma (24), Hepatocellular Carcinoma (40), ovarian cancer (10), Skin Carcinoma (100) prostate cancer (36), Mammary Tumor (33) Renal carcinoma (18), Pancreatic Cancer (80), and Rectal Cancer (10) [5–19]. After heterogeneity test (StataSE16.0), I^2^ = 86.9% and Q-test P < 0.05, suggesting a large heterogeneity among the literature selected for this study. Subgroup analysis of different tumor types showed that statistical heterogeneity (P < 0.05) still existed within certain subgroups (hepatocellular carcinoma, pancreatic cancer) (Fig. 3a). However, it was lower than before using subgroup analysis. The 15 papers in this study were subjected to meta-analysis using a random effects model. The results of the meta-analysis of random effects showed that the tumor volume was 3.80 points lower in the metformin-treated group than in the control group, and meta-regression analysis of this result was statistically significant (P < 0.05).

A total of 7 papers involving 292 experimental animals were included in this study [7, 20–25]. Five different cancer studies were included, lung cancer (74), hepatocellular carcinoma (18), colon cancer (80), ovarian cancer (20), and skin cancer (100). Heterogeneity test found I^2^ = 94.5%, Q test P < 0.05 (Fig. 3b). This indicates heterogeneity among the literature selected for this study. Subgroup analysis according to different cancer types revealed that heterogeneity within each subgroup was reduced compared to the previous subgroup but statistical heterogeneity was still present (p < 0.05). Therefore, the results of the combined analysis using a random effects model showed a 2.03 point reduction in the number of tumors in the metformin-treated group compared with the control group. Further meta-regression analysis showed that this result was statistically significant (p < 0.05).

A total of 4 papers were included in this study, in which a total of 92 experimental animals were reported [26–28], which involved 4 different cancer studies, namely Melanoma (12), ovarian cancer (20), pancreatic cancer (30), and endometrial cancer (30). A heterogeneity test revealed I^2^ = 93.6% and Q test P < 0.1. However, a subsequent sensitivity analysis (Fig. 3d) using Stata software revealed moderate heterogeneity (I^2^ = 75.5% and Q-test P < 0.1) after one of the pancreatic cancer-related publications [27] was excluded. The results showed a 5.71-point lower tumor weight in animals taking metformin compared to normal controls, which was found to be statistically significant (P < 0.05) using meta-regression analysis (Fig. 3c).

### Publication bias and sensitivity analysis

**Fig. 4:**
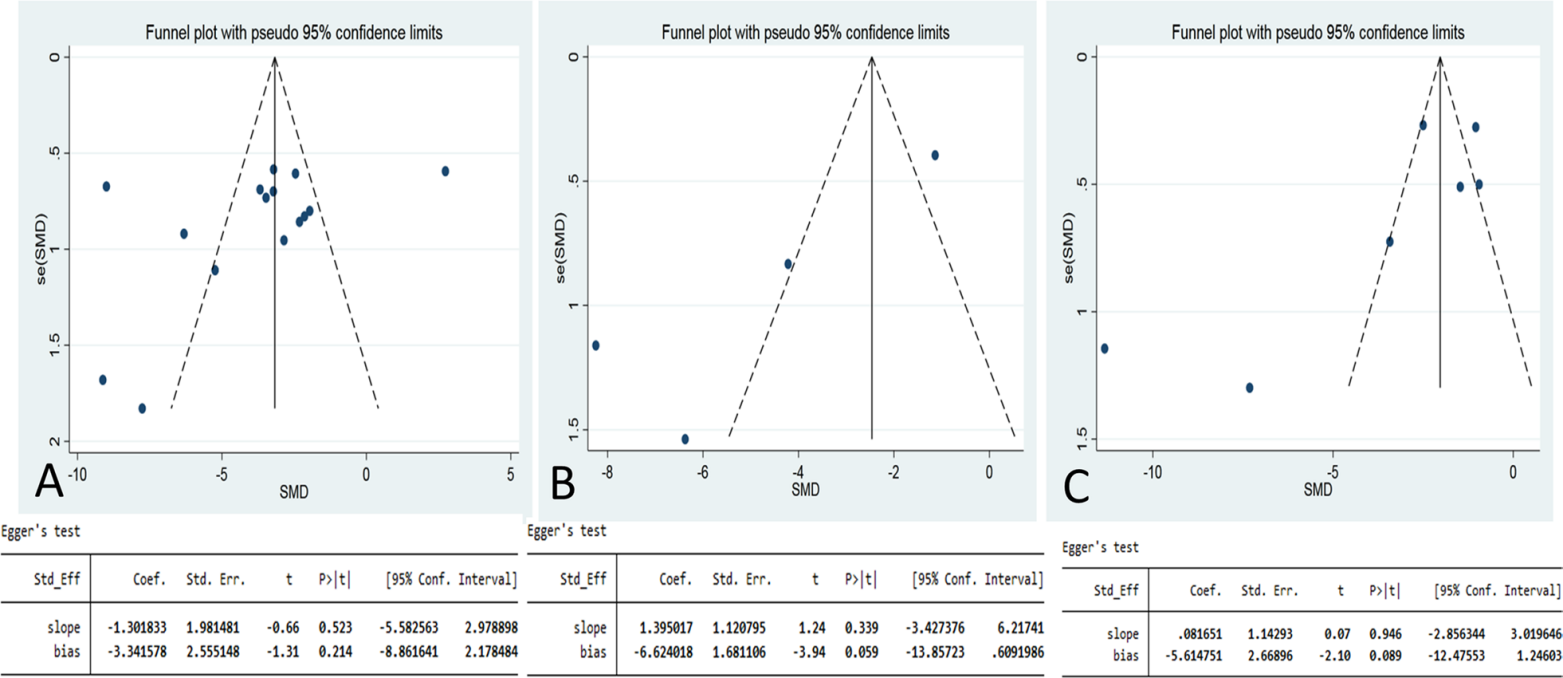
Funnel plot of the effect of metformin on tumor growth in different types of animal models. **a** Funnel plot of tumor volume studies. **b** Funnel plot of tumor weight studies. **c** Funnel plot of tumor number studies

According to the funnel plot shown above, the data distribution of the three indicators was basically symmetrical (tumor volume, number of tumors, and tumor weight), and the Eggers test showed (P < 0.05) that no significant publication bias was found (Fig. 4a–c).

## Conclusion

Metformin inhibits the proliferation of many types of cancer cells in vitro and in vivo. Several retrospective studies and prospective trials have also demonstrated that metformin reduces tumor incidence and mortality. Clinical trials using metformin as an adjuvant anticancer agent are being conducted extensively. A necessary generalization of existing studies can provide a basis for more in-depth research.

In this study, a comprehensive search of basic animal experimental studies on metformin for the treatment of various types of tumors was conducted through several databases using a systematic evaluation approach, and finally 25 English-language publications were included, and all 24 included studies were of high quality (Camarades' criteria of 5–8) (Table 1). In this study, quantitative synthesis of the study results using meta-analysis revealed that the tumor volume, weight and number of animals in 11 tumor models of different species were reduced to different degrees after metformin intervention, especially in the tumor volume of hepatocellular carcinoma (SMD = − 3.64, 95% CI − 4.73, − 2.54) and pancreatic cancer (SMD = − 3.46, 95% CI − 4.21, − 2.71) with tumor volume of lung cancer (SMD = − 3.10, 95% CI − 4.02, − 2.19).

By summarizing the existing animal experimental data, we found that metformin has therapeutic effects on different tumors, but the therapeutic effects are different. However, whether metformin can be used as an adjuvant antitumor agent and how it can be applied in clinical practice still needs to be confirmed by large-scale clinical studies, and it is expected to bring new hope for more tumor patients.

Although an extensive literature search was conducted for this study and no publication bias was found for the funnel plot and Eggers test, it cannot be excluded that some of the literature such as conferences and supplements are not available or some studies with negative results have not been published. The heterogeneity present in this study may have originated from different animal species (rats, mice) or different modeling methods, etc. Therefore, a random-effects model was used in this study for analysis and careful interpretation of the results. Most experimental animal models use healthy adult animals, while patients in clinical settings are often middle-aged and elderly with underlying diseases. Therefore, the results of animal experiments do not fully replicate the complex pathophysiology in the clinical setting. In conclusion, due to the limitations of basic experiments, the effect of metformin on tumor treatment still needs to be verified by further large-scale clinical trials.

## Data Availability

The datasets used or analysed during the current study are available from the corresponding author on reasonable request.
